# Age-Related Gray and White Matter Changes in Normal Adult Brains

**DOI:** 10.14336/AD.2017.0502

**Published:** 2017-12-01

**Authors:** Farnaz Farokhian, Chunlan Yang, Iman Beheshti, Hiroshi Matsuda, Shuicai Wu

**Affiliations:** ^1^College of Life Science and Bioengineering, Beijing University of Technology, Beijing, 100022, China; ^2^Integrative Brain Imaging Center, National Center of Neurology and Psychiatry, Kodaira, Tokyo Japan

**Keywords:** aging, gender, MRI, voxel-based morphometry, brain volume

## Abstract

Normal aging is associated with both structural changes in many brain regions and functional declines in several cognitive domains with advancing age. Advanced neuroimaging techniques enable explorative analyses of structural alterations that can be used as assessments of such age-related changes. Here we used voxel-based morphometry (VBM) to investigate regional and global brain volume differences among four groups of healthy adults from the IXI Dataset: older females (OF, mean age 68.35 yrs; n=69), older males (OM, 68.43 yrs; n=66), young females (YF, 27.09 yrs; n=71), and young males (YM, 27.91 yrs; n=71), using 3D T1-weighted MRI data. At the global level, we investigated the influence of age and gender on brain volumes using a two-way analysis of variance. With respect to gender, we used the Pearson correlation to investigate global brain volume alterations due to age in the older and young groups. At the regional level, we used a flexible factorial statistical test to compare the means of gray matter (GM) and white matter (WM) volume alterations among the four groups. We observed different patterns in both the global and regional GM and WM alterations in the young and older groups with respect to gender. At the global level, we observed significant influences of age and gender on global brain volumes. At the regional level, the older subjects showed a widespread reduction in GM volume in regions of the frontal, insular, and cingulate cortices compared to the young subjects in both genders. Compared to the young subjects, the older subjects showed a widespread WM decline prominently in the thalamic radiations, in addition to increased WM in pericentral and occipital areas. Knowledge of these observed brain volume differences and changes may contribute to the elucidation of mechanisms underlying aging as well as age-related brain atrophy and disease.

In the human brain, magnetic resonance imaging (MRI) has revealed morphometric brain changes due to development and aging [1-3]. Various studies show that there is a significant link between age and cognitive functions such as memory, language, attention, thinking, and executive skills [4-6]. The many investigations of the human brain over the past several decades have broadened our understanding of the brain and contributed to the monitoring of clinical treatment effects in many brain diseases in aging individuals, including Alzheimer's disease, Parkinson's disease, schizophrenia, dementia, depression, and multiple sclerosis. In addition, several research groups have investigated the effects of age on gray matter density [7], cortical thickness [8], white matter signal abnormalities [9], and alterations in structural and functional brain systems [10].

In the present study, we investigated the differences in global and regional brain volume alterations between young and older adults with respect to gender. We used structural magnetic resonance imaging (sMRI) data because of its advantages (including good tissue contrast and excellent spatial resolution without radiation exposure), which are lacking in positron emission tomography and single photon emission computed tomography modalities. The location-specific measures derived from sMRI are influential biomarkers for assessments of brain volume alterations.

Voxel-based morphometry (VBM) was developed as an advanced method for performing group-wise comparisons of sMRI scans [11]. Briefly, VBM assesses whole-brain structures with voxel-by-voxel comparisons that can be used to compare tissue concentrations or volumes between subject groups to distinguish structural alterations [12]. The VBM technique has been widely used to assess the gray matter (GM) and white matter (WM) alterations in various brain diseases such as Alzheimer's disease [13-15], Parkinson's disease [16], and epilepsy [17]. We used the VBM technique in the present study to determine the overall and regional brain volume differences among healthy male and female young (~late 20s) and older (~late 60s) individuals.

**Figure 1.**
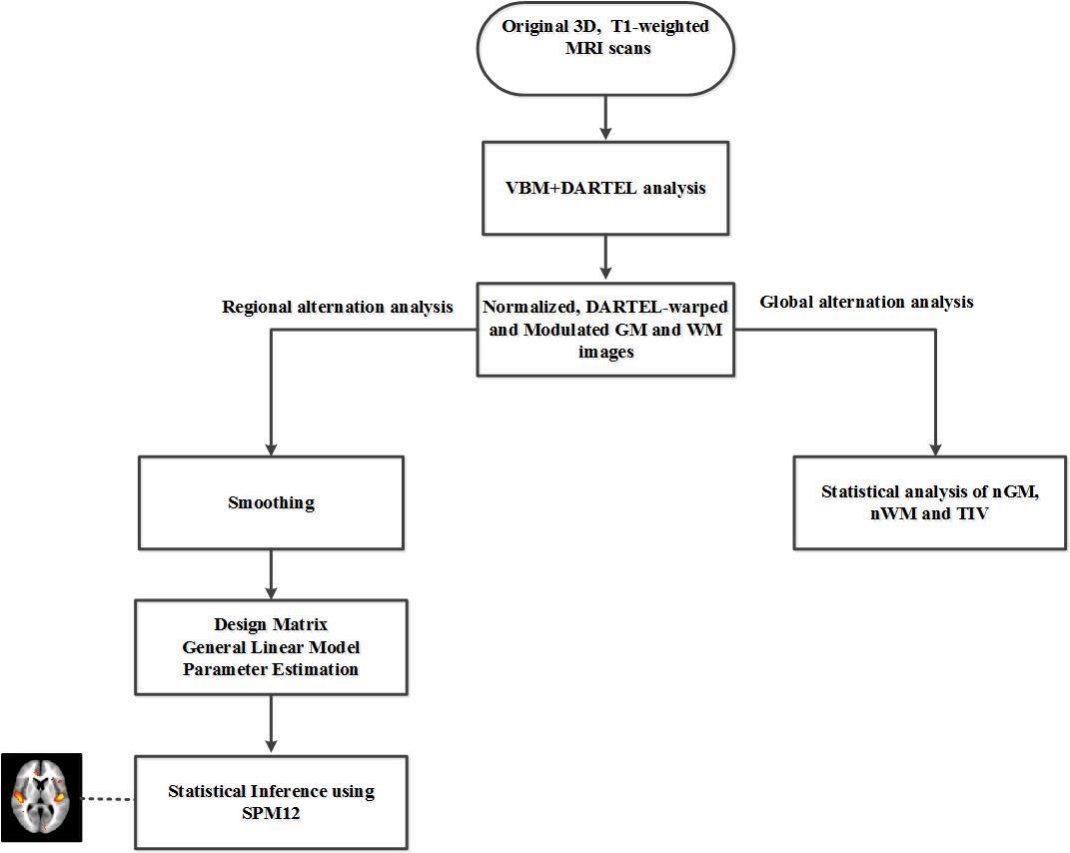
The general structure of proposed analysis procedure.

## MATERIALS AND METHODS

### Image acquisition and subjects

The data in the context of the present study were acquired from the publicly accessible IXI Dataset (brain-development.org/ixi-dataset/). The MRI scans were acquired from three sites with 1.5 and 3T scanners (FoV=256 mm×256 mm, matrix size=0.9375×0.9375×1.2 mm^3^). Details of the IXI data and scan parameters are at: (http://biomedic.doc.ic.ac.uk/brain-development/index.php?n=Main.Datasets).

As summarized in Table 1, we selected a total of 277 healthy subjects from the IXI Dataset and categorized them into the following four age/gender groups: older females (OF, mean age 68.35 yrs; n=69), older males (OM, 68.43 yrs; n=66), young females (YF, 27.09 yrs; n=71), and young males (YM, 27.91 yrs; n=71). There was no significant difference in age between the OF and OM groups or between the YF and YM groups.

**Table 1:** The characteristics of the four groups of healthy subjects from the IXI Dataset

	Older females (n=69)	Older males (n=66)	Young females (n=71)	Young males (n=71)
Age (yrs) Range	68.35±5.80 (60-86)	68.43±6.21 (60-86)	27.09±3.59 (20-34)	27.91±3.79 (20-34)

All data are mean±standard deviation (SD).

### Methodology

Figure 1 illustrates the major components of the methodology used in this study, including the acquisition of MRI scans, the VBM analysis, and the statistical analysis of brain volumes in the young and older groups with regard to gender.

The MRI preprocessing was performed using the Computational Anatomy Toolbox (CAT12; http://dbm.neuro.uni-jena.de/cat) [18] and Statistical Parameter Mapping (SPM) software ver. 12 (http://www.fil.ion.ucl.ac.uk/spm). Briefly, all MRI scans were segmented into WM, GM, and cerebrospinal fluid (CSF) components using the unified segmentation model [19], and then modulated and normalized into a Montreal Neurological Institute (MNI) template.

To provide a more regional and nonlinear deformation and to increase the intersubjective alignment of the MRI scans, we applied the DARTEL approach in the spatial normalization stage. The DARTEL approach helps optimize the sensitivity of such analyses by using the Levenberg-Marquardt strategy [20]. In addition, the DARTEL approach can provide more precise spatial normalization to the template compared to the conventional algorithm [21-23]. The details of a comparison of the DARTEL approach and the standard registration methods were as described [24].

In this study, we used the GM and WM images. Finally, with the use of an 8-mm full width at half maximum (FWHM) Gaussian isotropic kernel, the segmented GM and WM images were spatially smoothed. The smoothed, modulated, DARTEL-warped and normalized GM and WM modalities were used for our statistical analysis through a flexible factorial in the SPM12 program. Regional GM and WM alterations were generated by a voxel-based analysis over the whole brain. The absolute threshold for masking was adjusted to 0.2 to avoid possible edge effects between GM and WM or CSF. Group comparisons were assessed with the use of a family-wise error (FWE) at a threshold of *p*<0.05, corrected for multiple comparisons; statistical significance was determined using an extent threshold of 100 adjacent voxels.

In addition, to explore the regional GM and WM differences, we identified the global brain tissue volumes in the two older subject groups versus the two young groups with regard to gender. In this manner, we calculated the absolute GM volume (GMV), the absolute WM volume (WMV), and the absolute CSF volume as well as the total intracranial volume (TIV) of each subject. The TIV was calculated as the sum of the GMV, WMV, and CSF volumes. In order to correct for variation in the subjects' head sizes, we also calculated the normalized GM (nGM) and normalized WM (nWM) by dividing the individual subjects' GMV and WMV values by each subject's respective TIV value.

### Statistical analysis

To assess the influence of two independent variables (age, gender) on global brain variables, we performed a two-way analysis of variance (ANOVA). Two levels of age (young and older) and gender (female and male) were examined. Between-group differences in global brain variables among or between groups were examined by an ANOVA followed by Tukey's multiple comparison test. We used the Pearson correlation test to investigate the association between brain volume changes and age. All statistical analyses were performed using Statistical Package for Social Sciences software (SPSS, ver. 16.0; SPSS, Chicago, IL) with *p*<0.05 as the significance level.

## RESULTS

### Global differences in brain volume

Whole-brain analyses help provide a reliable indication of total brain volume differences (in accord with age and gender) which occur in the entire brain. As described above in Section 2.2, we calculated the nGM and nWM in order to correct for variations in head size. Here we report the statistical results related to the influence of age and gender on global brain tissue volumes (i.e., the nGM, nWM and TIV) as well as the correlations between age and global brain tissue volume differences with respect to gender.

**Figure 2.**
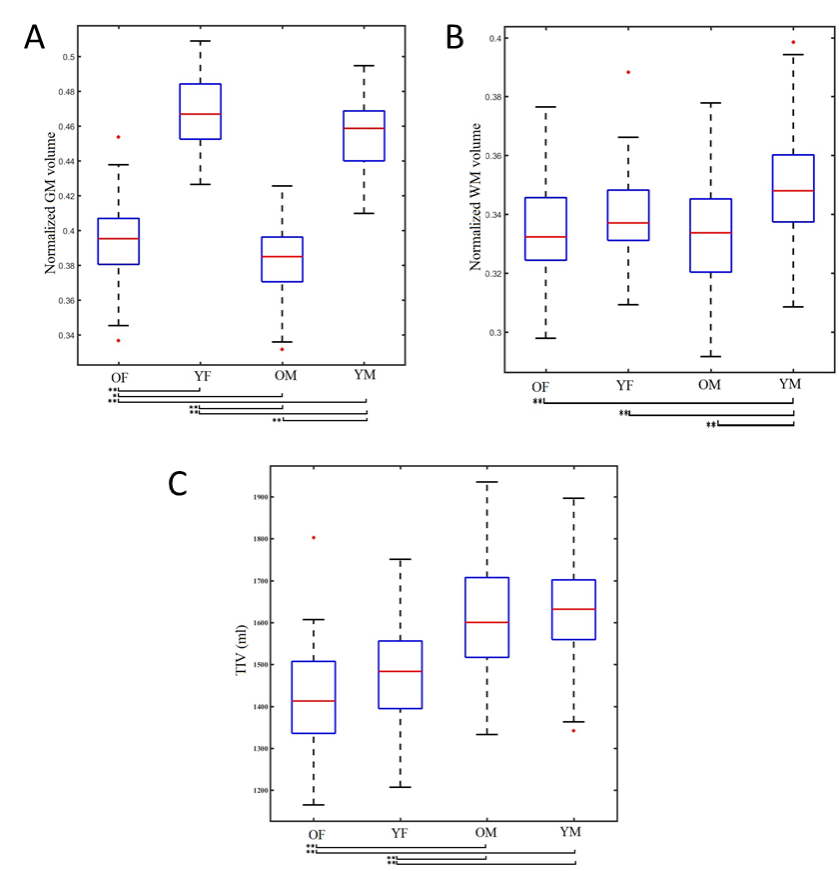
The box plots for the OF, YF, OM, and YM subjects The box plots for the OF, YF, OM, and YM subjects' (**A**) normalized GM, (**B**) normalized WM and (**C**) total intracranial volume (TIV). Significance was determined by an ANOVA followed by Tukey's post-hoc test. *p<0.05, **p<0.001.

#### The influence of age and gender on global brain volumes

Table 2 presents the ranges of the whole-brain tissue volumes in the four subject groups. Regarding the nGM, the main effect of the age level yielded an F-ratio of F[1,273]=894.60, *p*<0.001, indicating a significant difference in nGM between the young subjects (mean [M]=0.462, SD=0.020) and the older subjects (M=0.389, SD=0.021). The main effect of gender yielded an F-ratio of F[1,273]=25.25, *p*<0.001, revealing a significant difference in nGM between the female (M=0.432, SD=0.042) and male subjects (M=0.420, SD=0.040). The interaction effect between age and gender on nGM was not significant: F[1,273]=0.079, *p*=0.77.

In a direct comparison of the YF versus OF subjects, the average values of nGM were 0.468±0.022 and 0.399±0.025 (mean difference [MD]=0.073, *p*<0.001), whereas the average nGM values of the YM and OM subjects were 0.455±0.012 and 0.383±0.016 (MD=0.072, *p*<0.001), respectively. Figure 2A shows the normalized GM box plots for the four subject groups.

Regarding the nWM, the main effect of age yielded an F-ratio of F[1,273]=30.39, *p*<0.001, indicating a significant difference between the young subjects (M=0.344, SD=0.017) and the older subjects (M=0.333, SD=0.016). The main effect of gender yielded an F-ratio of F[1,273]=5.90, *p*<0.05, demonstrating a significant difference in nWM between the females (M=0.336, SD=0.015) and males (M=0.341, SD=0.019). There was a significant interaction of age and gender on the nWM: F[1,273]=9.52, *p*<0.05.

In our direct comparison of the YM and OM subjects, the groups' average nWM values were 0.349±0.013 and 0.332±0.017 (MD=0.017, *p*<0.01), respectively. However, there was no significant difference in the average nWM values of the YF and OF groups. Figure 2b shows the normalized WM box plots of the four age×gender groups.

Regarding the TIV, the main effect of age showed an F-ratio of F[1,273]=5.54, *p*<0.05, i.e., a significant difference between the young subjects (M=1548.60, SD=142.19) and the older subjects (M=1512.40, SD=149.93). The main effect of gender had an F-ratio of F[1,273]=127.30, *p*<0.001, revealing a significant difference in TIV between the female (M=1449.80, SD=119.08) and male subjects (M=1613.90, SD=124.77). The interaction effect was not significant: F[1,273]=0.78, *p*=0.37.

In our direct comparisons between the YF versus OF subjects and the YM versus OM subjects, there were no significant differences in the average of TIV values. This absence of significant differences in TIV showed that the two female groups (YF and OF) were comparable with respect to head size, as were the two male groups (YM and OM). Figure 2c shows the TIV box plot for each group.

**Table 2:** The range of global volume measurements for the young and older female and male subjects

	Older females (n=69)	Young females (n=66)	Older males (n=71)	Young males (n=71)
GM (ml)	562.59±50.08	689.66±59.77	614.28±60.11	739.58±56.97
WM (ml)	476.56±44.65	499.70±52.09	533.98±57.17	568.6425±53.71
TIV (ml)	1425.90±108.93	1473.12±124.58	1602.91±132.94	1624.20±116.68
nGM	0.399±0.02	0.468±0.02	0.383±0.01	0.455±0.01
nWM	0.334±0.01	0.339±0.01	0.332±0.01	0.349±0.01

All data are mean±SD. GM: gray matter; WM: white matter; TIV: total intracranial volume; nGM: normalized gray matter; nWM: normalized white matter.

#### The correlation between the global brain tissue volume changes and age

**Figure 3.**
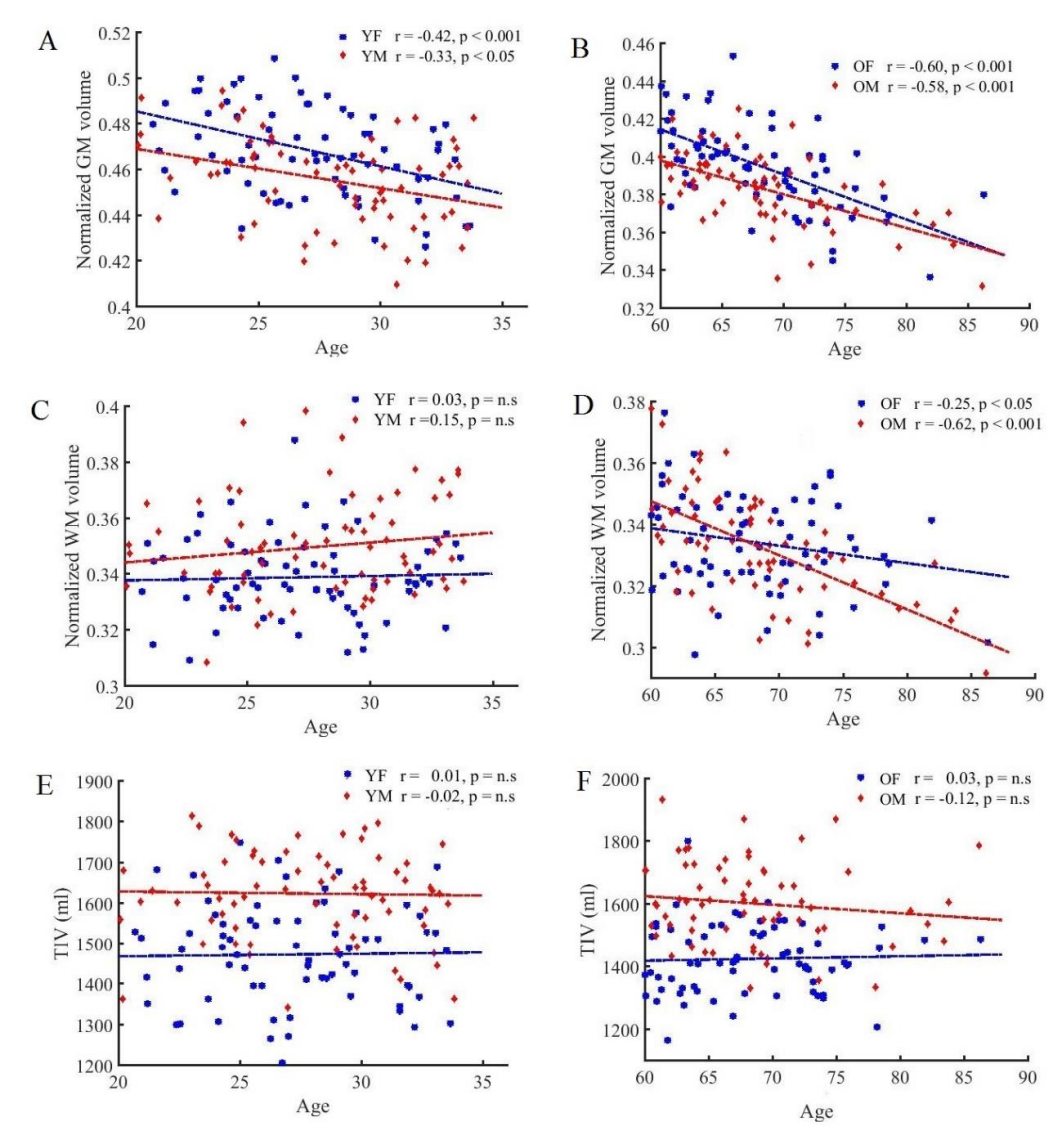
Plots of brain volumes vs. age in the young and older groups with respect to gender (**A**) normalized GMV in the young subjects, (**B**) normalized GMV in the older subjects, (**C**) normalized WMV in the young subjects, (**D**) normalized WMV in the older subjects, (**E**) TIV in the young subjects, and (**F**) TIV in the older subjects.

To determine the effect of age on brain tissue volume changes, we estimated the correlation between age and global brain tissue volumes among the subject groups. Figure 3a–f illustrates the results of the correlation analysis of nGM, nWM, and TIV values in accord with age and gender.

Regarding the nGM (Fig. 3A, B), there was a strong negative correlation between age and nGMV in the young subjects (females: *r* (n=71)=-0.42, *p*<0.001; males: *r* (n=71)=-0.33, *p*<0.001) and in the older subjects (females: *r* (n=69)=-0.60, *p*<0.001; males: *r* (n=66)=-0.58, *p*<0.001). In the comparison of the nGM changes, the statistical analysis revealed a significant linear reduction with age in the older subjects as well as in the young subjects. In comparison to the males, this decrease was steeper in the females in both the older and young groups.

Regarding the nWM (Fig. 3C, D), we observed a weak interaction between nWM and age in the young subjects (females: *r* (71)=0.03, *p*=ns; males: *r* (71)=0.15, *p*=ns). Notably, the Pearson correlation test revealed a significant negative interaction between nWM and age in the older subjects (females: *r* (69)=-0.25, *p*<0.05; males: *r* (66)=-0.62, *p*<0.001), and this decrease was significantly steeper in the males compared to the females. There was no significant relationship between TIV and age in the various groups (*p*=ns) (Fig. 3E,F).

### Regional differences in brain volume

#### GM regional differences

**Figure 4.**
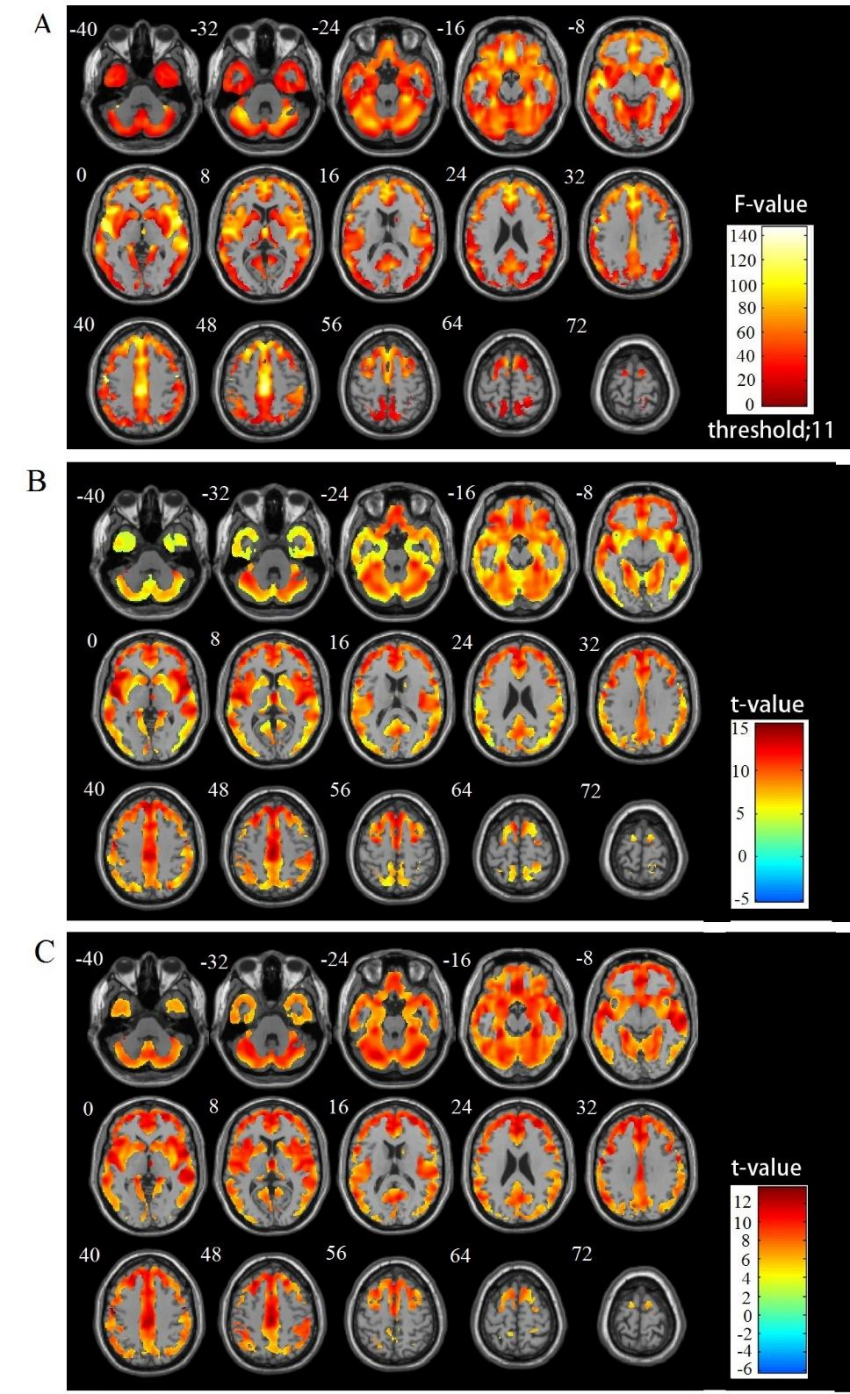
Group comparisons of GM volume alterations by VBM using SPM12 and DARTEL (FWE corrected at *p*<0.05 with extend threshold K=100) (**A**) F-test results for the four groups, (**B**) OF vs. YF, and (**C**) OM vs. YM. Warm and cool color scales show negative and positive correlations with age and volume, respectively

Figure 4 shows the results of the group comparison of GM volume between the older and young subjects with respect to gender as well as the F-test results for the four groups. The VBM analysis revealed a diffuse and age-related reduction in the GM volume prominently in the frontal, insular, and cingulate cortex in both genders. In contrast, the occipitoparietal areas, medial temporal structures, and subcortical gray matter were relatively spared the age-related reduction in both genders. No significant interaction of age×gender regarding the reginal GM volume was observed. The reverse contrast showed no significant GMV reduction in the young subjects compared with the aging subjects.

#### WM regional differences

**Figure 5.**
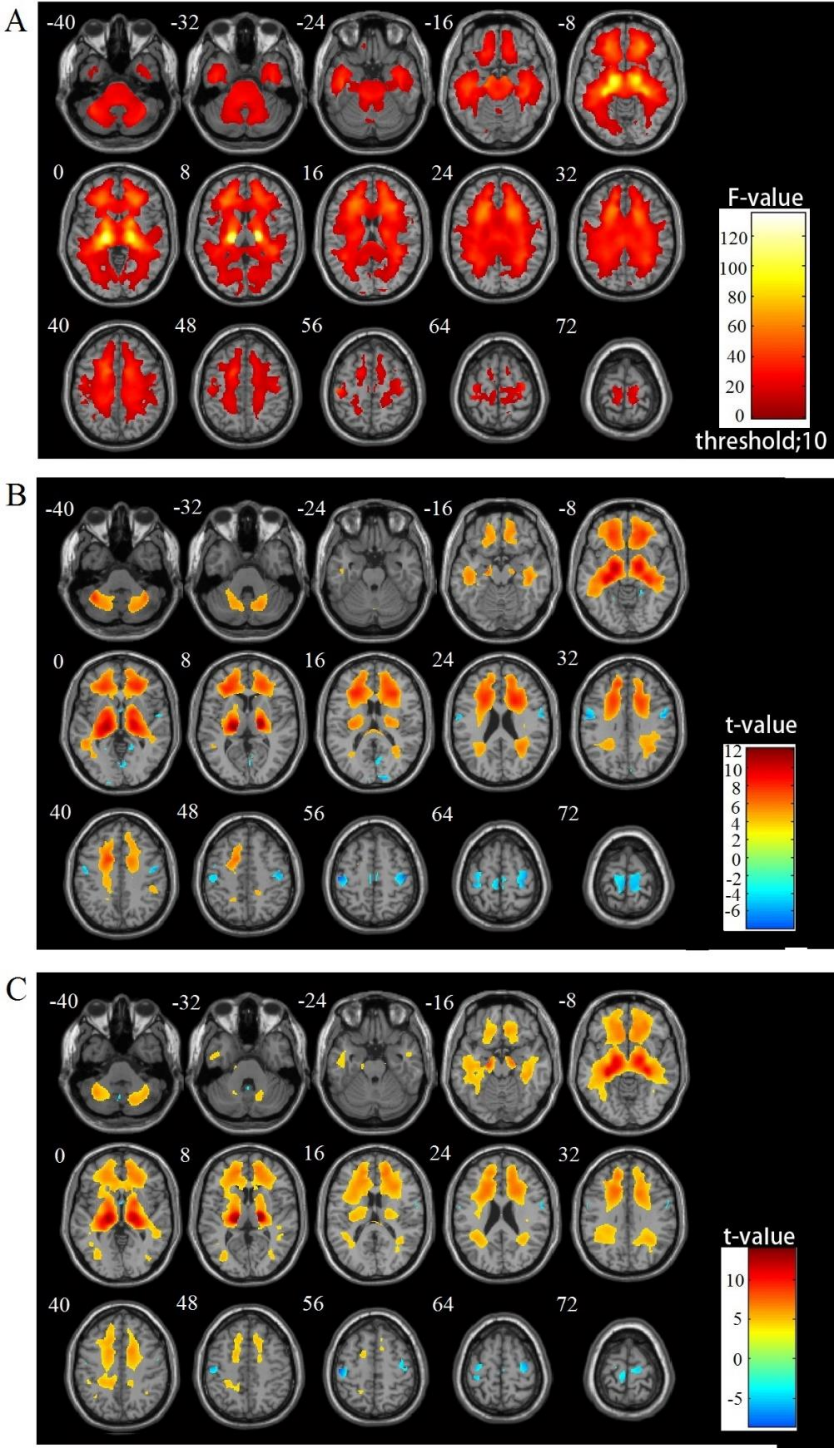
Group comparisons of WM volume alterations by VBM using SPM12 and DARTEL (FWE corrected at *p*<0.05 with extend threshold K=100) (**A**) F-test results of the four groups, (**B**) OF vs. YF, and (**C**) OM vs. YM. Warm and cool color scales show negative and positive correlations with age and volume, respectively.

Figure 5 illustrates the results of the group comparisons of WM volume between the older and young subjects by gender as well as the F-test results among the four groups. The VBM analysis revealed an age-related decline in the WM volume, prominently in the thalamic radiations. In contrast, there was an increase in WM volume in the paracentral and occipital areas in both the female and male subjects. No significant interaction of age×gender was observed regarding reginal WM volume.

## DISCUSSION

Our present findings indicate significant differences in the regional and global brain tissue volumes between our young and older subjects with respect to their gender. Further investigations of brain tissue volume differences between young and older individuals could lead to a better understanding of the normal aging process and clarify the pathology of age-associated neurological diseases. Several studies investigated the influence of aging on brain structures over time [8, 25-30]. Our present findings showed different global and regional patterns of GM and WM differences in healthy young and older individuals with respect to gender, as discussed in detail below.

### GM differences

In our global GM analysis, the two-way ANOVA revealed significant influences of age and gender on global nGM volumes, in that the young subjects had superior total normalized GM volumes compared to the older subjects (i.e., YF and YM vs. OF and OM), and the female subjects showed higher nGM compared to the males (YF and OF vs. YM and OM).

In the direct comparisons between the YF and OF subjects and between the YM and OM subjects, we observed superior total nGM volumes in the young females and young males compared to the older females and aging males, respectively (Fig. 2A). In addition, the correlation test showed that the normalized GM volume decreased linearly with age; this decrease was steeper in the females not only among the older subjects but also in the young group (Fig. 3A, B). Regarding the regional GM changes, we observed significant GM volume reductions in the frontal, insular, and cingulate cortices and a preservation of GM volume reductions in the occipitoparietal areas and subcortical regions among the older subjects compared to the young subjects of both genders.

These findings are generally in line with those of other studies which reported a linear negative association between GM volume and age for most cortical regions, prominently in the frontal and insular areas [26, 29-31]. Our results are also in agreement with other studies that demonstrated a preservation of the GM volume in specific structures (such as limbic and paralimbic brain structures) over the aging process[29, 31-33]. Consequently, it can be hypothesized that there are no significant alterations in limbic structures in older individuals with advancing age unless the alterations are due to specific neuropathological processes such as those related to cerebrovascular disease [34] or Alzheimer's disease [35].

### WM differences

In our global WM analysis, the two-way ANOVA showed significant influences of age and gender on global nWM volumes, in that the young subjects showed higher nWM volumes compared to the older subjects (i.e., YF and YM vs. OF and OM), and the males showed slightly higher nWM volumes compared to the females (YM and OM vs. YF and OF).

In our direct comparison of the YF versus OF and YM versus OM groups, we observed a significant difference in nWM volumes between the older and young subjects among the males. Conversely, the difference in normalized WM volumes between the older and young subjects among the females was not significant (Fig. 2b). The reason(s) for this discrepancy between the genders are not clear, but may be related to the influence of sex hormones [36, 37].

Our analyses also demonstrated that there was no significant correlation between nWM volume changes with age in the young subjects (Fig. 3C) of both genders. The correlation test showed a significant negative association between nWM volume changes with age only in the older group for both genders, and this was significantly steeper in the male subjects (Fig. 3D).

With respect to regional WM differences, we observed a widespread reduction of WM volume prominently in the thalamic radiations. In contrast to some studies of healthy aging which describe only a decrease of WM in normal aging [31, 38, 39], we also observed significant WM increases in the pericentral and occipital areas among our older subjects compared to the young subjects, of both genders. The reason for this increase may be due to the ongoing maturation of the white matter during normal aging [40]. Our findings are broadly consistent with previous studies [20, 40] that reported a linear negative WM volume reduction associated with advancing age in anterior thalamic radiations, the internal capsule, cerebral peduncle, cerebellum, and external capsule among older subjects and a slight WM volume increase in bilateral corona radiata in the older subjects.

In future research, a diffusion tensor imaging analysis [41] between young and aging subjects with respect to gender should be considered. Another priority will be to use an individual network analysis [42] to determine the structural brain network differences between young and aging individuals with the consideration of gender.

## Conclusion

In conclusion, we assessed regional and global brain tissue volume differences by conducting a VBM analysis of healthy young and older subjects of both genders. Our statistical analyses revealed different patterns of age-associated alterations in both gray and white matter volumes in the young and older subjects. On the global level, we examined the effects of age and gender on normalized gray and white matter volumes as well as the total intracranial volume. We also explored the association between brain tissue volume changes with age in young and older subjects with respect to gender. We investigated the regional gray and white matter volume changes that had occurred in the brains of healthy subjects with age compared to those of young subjects, in both genders. Our findings indicate that there is a significant effect of brain volume changes during the aging process. Thus, the knowledge of brain volume changes and differences may contribute to a better understanding of the roots of health and disease in the later stages of life [43, 44].
